# ChatGPT4.o Geriatrics Knowledge Competency and Its Evaluation by Geriatricians

**DOI:** 10.1093/geroni/igaf122.1182

**Published:** 2025-12-31

**Authors:** Iriana Hammel, Natasha Resendes, Dominique Tosi, Gokhan Demir, Huai Cheng

**Affiliations:** Miami VA Medical Center, Miami, Florida, United States; Miami VA GRECC, Miami, Florida, United States; Miami VA Heathcare System, Miami, Florida, United States; University of Miami, Coral Gables, Florida, United States; Minneapolis VA Hospital, Minneapolis, Minnesota, United States

## Abstract

ChatGPT has passed USMLE and other medical knowledge exams, demonstrating competence in the medical field. It is less studied in Geriatric Medicine specifically. This study aimed to evaluate the geriatric competency of ChatGPT4.o by examining its performance on the validated UCLA Geriatrics knowledge tests, comparing its performance with that of trainees and exploring whether geriatricians agree with ChatGPT 4.o’s performance. 18 UCLA Geriatrics knowledge questions were answered. “Correct answer” was graded as 1, “incorrect answer” as -1 and “don’t know” as 0. The total score was between -18 to + 18. Test scores were calculated to compare ChatGPT and trainees (medical students, internal medicine residents and Geriatric medicine fellows) from previously published studies. ChatGPT4.o responses were evaluated by participants and graded on a Likert scale of 1-5 (1=strongly disagree, 5=strongly agree). Score of Geriatric knowledge by ChatGPT 4.0 was 18, higher than all trainees (9.9, 9.5, 13.6, 13,2, 14.7, 14.9. and 17.5 for MS1, MS2, MS3, PGY 1, 2 and 3 IM residents, and Geriatrics fellows respectively). Six geriatricians (four faculty and two fellows) rated its performance at 4.2 on a 1-5 Likert scale. In conclusion, ChatGPT4.o outperformed the trainees in Geriatric knowledge tests. Geriatricians were in agreement with ChatGPT4.o performance. It suggests ChatGPT4.o has competency in Geriatric knowledge. Concurrent control trainees’ testing is underway, as their scores may differ than those of the historical controls’ scores due to the trainees’ own use of artificial intelligence.

